# Supplementary material for: The splicing factor XAB2 interacts with ERCC1-XPF and XPG for R-loop processing
Source: Nat Commun. 2021 May 26;12:3153. doi: 10.1038/s41467-021-23505-1 (PMC8155215; doi:10.1038/s41467-021-23505-1)
Supplement: Supplementary file 1 — Supplementary Information [file 41467_2021_23505_MOESM1_ESM.pdf]

Supplementary information

Supplementary figure legends

Figure S1. XAB2-bound protein partners involved in DNA repair and validation of XAB2 silencing and putative RNA binding activity. (A). List of significantly over-represented biological processes of 636 shared XAB2-bound proteins. (B). bXAB2 pulldowns followed by western blot analysis with DDB1 or XPA in UV-irradiated (10J/m2) and control primary MEFs. (C). Immunofluorescence detection of XAB2 with UV-induced CPDs; white arrowheads indicate the immunodetection of XAB2 and CPDs, respectively (D). Immunofluorescence detection of XAB2 with γH2Ax in UV-irradiated HEPA cells (10J/m2). (E) Western blot analysis of XAB2 protein levels in si*Xab2* cells and si Scramble (Scrb) cells at 48 hours (h) post-transfection. (F). % of live si*Xab2* cells (Trypan blue inclusion) at the indicated time points post transfection; the red dotted line indicates the 100% survival. (G). Mean number (no) of colonies of si*Xab2* cells at seven days post-transfection derived from 6 independent experiments. (H-I). Western blot analysis of RNAPII, DDB1, XPA and ERCC1 in siScrb and si*Xab2* cells at 48h post-transfection.. (J).mRNA levels and (K) Pre-mRNA levels of transcripts with retained introns in untreated and 30 or 60μM isoG treated wt MEF cells at 12 hours post-inhibitor treatment. (L). Multiple alignment between the HCF107 RNA binding and U4, U5, U6 snRNA sequences; red nucleotides represent core binding sequence. The putative conserved nucleotides between snRNAs and HCF107 RNA binding sequence are highlighted in bold. The alignment includes the 5'biotin labelled oligo (XAB2RBS) and its mutated version (mutRBS). (M-N). Gel mobility shift assay showing 100pmoles of the biotin labelled probes XAB2RBS and mut RBS after incubation: (M) without (lane 1) or with increasing amounts of the eluate deriving from XAB2 IP or IgG IP from nuclear

extracts and(N)without (lane 1) or with increasing amounts of recombinant human XAB2. Gel mobility shift assay reactions were analyzed concomitantly on polyacrylamide gels. The images shown on Figure S1B-I are representative of experiments that were repeated three times. All scatter and bar blots are presented as mean ± SEM. *p* values were calculated by two tailed Student's t test. Scale bars, 5 µm. Source data provided as a Source Data file.

Figure S2. XAB2 is required for pre-mRNA splicing (A). A table summarizing the data from all alternative splicing events considered and the differentially spliced events in si*Xab2* and siScrb HEPA cells. Pdiff: probability of differential splicing (B). Distribution of all alternative splicing events considered and (C) differentially spliced events in si*Xab2* and si Scramble (Scrb) HEPA cells. (D). Volcano plots of all exon skipping events and (E). Intron retention events considered in si*Xab2* and si Scramble (Scrb) HEPA cells. Intron retention (IR); Exon Skipping (EX); Alternative 3' spliced sites (Alt3'); Alternative 5' spliced sites (Alt5'); Percent spliced-in (PSI). (F). A table depicting the data of all alternative splicing events considered and the differentially spliced events in si*Xab2* and si Scramble (Scrb) mESCs. Pdiff: probability of differential splicing (G). Distribution of all alternative splicing events considered and (H) differentially spliced events in si*Xab2* and si Scramble (Scrb) mESCs. (I). Volcano plots of all exon skipping events and (J). Intron retention events considered in si*Xab2* and siScrb mESCs.

Figure S3 XAB2 binding on pre-mRNAs and spliceosome disassembly upon UV irradiation. (A). Over-representation of biological processes that are significantly affected by alternative splicing in si*Xab2* HEPA cells. Intron retention (IR); Exon Skipping (EX); Alternative 3' spliced sites (Alt3'); Alternative 5' spliced sites (Alt5'); Percent spliced-in (PSI); FDR: False Detection Rate; F.E: Fold Enrichment;

GC: Gene count. . (B). Over-representation of biological processes that are significantly affected by alternative splicing in si*Xab2* mESCs. (C-D). bXAB2 RNA pulldowns for the indicated pre-mRNAs in P15 livers (E). Pre-mRNA levels of transcripts with retained introns in si2*Xab2* and siScrb HEPA cells at 48h post-transfection. (F). Chromatin-associated snRNAs quantified by Q-PCR and normalized to Neat1 lncRNA in wt. MEFs and MEFs at different time points after exposure to UV. (G). Prp3 RNA pulldowns on U4 and U6 snRNA in wt. MEFs and MEFs at different time points after exposure to UV. (H). XAB2 and Aquarius RNA pulldown on U4 and U6 snRNA in wt. MEFs and MEFs at different time points after exposure to UV. RIP signals are shown, either as fold enrichment (F.E) of % input of antibody/bXAB2 over % input of control antibody (IgG)/BirA or as of % input of bXAB2 over % input of BirA. All scatter and bar blots are presented as mean ± SEM. Error bars indicate S.E.M. among three biological replicates in all cases. *p* values were calculated by two tailed Student's t test, unless otherwise indicated. Source data provided as a Source Data file.

Figure S4. Transcription inhibition displaces XAB2 from spliceosome. (A). Pre-mRNA levels of UV-irradiated cells compared to untreated controls. (B). Immunofluorescence detection of RNA synthesis recovery in wt. cells treated with 5-Bromouridine (BrU) at different time points after UV irradiation (10J/m2) (unt: n=185 cells/3 independent experiments, 2h: n=245 cells/3 independent experiments, 6h: n=170 cells/3 independent experiments, 24h: n=125 cells/3 independent experiments). (C). RNA immunoprecipitation of XAB2 on U4 in wt. cells and IsoG-treated cells (D-E). RNA immunoprecipitation of XAB2 on U4/U6 snRNAs and on selected pre-mRNAs in wt. cells and cells treated with the RNAPII transcription elongation inhibitor 5, 6-Dichloro-1-β-D-ribofuranosylbenzimidazole (DRB) and the

RNAPI- and II-mediated transcription initiation inhibitor tryptolide (TPL). In the case of pre-mRNAs, the *p* value was calculated by one-tailed Student's t-test. (F). bXAB2 RNA pull downs on pre-mRNAs in P15 bXAB2 or bXAB2;*Csb*$^{m/m}$ livers. (G). Representative images and quantification of RNA:DNA hybrid immunofluorescence detection in siScrb and si*Xab2* MEFs without UV-irradiation or 8 hours post-UV irradiation. Graph depicts mean S9.6 fluorescence intensity per nucleus from three representative experiment (scrb: n=205 cells/3 independent experiments, scrb + UV: n= 250 cells/3 independent experiments, si: n= 125 cells/3 independent experiments, si + UV: n=100 cells/ 3 independent experiments). (H). Immunofluorescence detection of γH2AX and 53BP1 (white arrowheads) in siScrb and si*Xab2* MEFs with or without UV-irradiation. The graph represents the % of γH2AX+ 53BP1+ cells (scrb: n= 506 cells/3 independent experiments, scrb + UV: n=414 cells/3 independent experiments, si: n= 443 cells/3 independent experiments, si + UV: n=425 cells/ 3 independent experiments). (I) RNA:DNA hybrid immunofluorescence detection in untreated and IsoG-treated MEFs. Higher magnifications of cells in white box and quantifications are presented in Figure 6B. RIP signals are shown as fold enrichment (F.E) of % input of antibody or bXAB2 over % input of control antibody (IgG) or BirA. All scatter and bar blots are presented as mean ± SEM. Error bars indicate S.E.M. among three biological replicates in all cases. *p* values were calculated by two tailed Student's t test, unless otherwise indicated. Scale bars, 15 μm. Source data provided as a Source Data file.

Figure S5. UV irradiation leads to XAB2 release from R-loops (A) Basal levels of R loops were monitored and quantitated by immunofluorescence with S9.6 antibody in si*Scrb* and si*Xab2* transfected B6 MEFs. Higher magnifications of cells in white box are presented in Figure 6C in the case of si1*Xab2* or in insets in the case of si2*Xab2*.

Quantifications of immunofluorescence are shown in figure 6C. (B). DRIP analysis of tRA-responsive (*Rarb2*, *Sstr4, Fibin* and *Stra6*) and non-responsive (*ChordC*) gene promoters in untreated, tRA-treated, tRA and illudin or DRB treated MEFs, as well as in MEFs exposed to UV with or without tRA treatment. (C) DRIP analysis of tRA-responsive (*Rarb2*, *Stra6*) gene bodies using the S9.6 antibody against RNA-DNA hybrids in tRA-, tRA- and DRB-treated MEFs, as well as tRA-treated MEFs exposed to UV irradiation and UV-irradiated MEFs (D). bXAB2 ChIP signals from tRA- or illudinS/tRA-treated BirA and bXAB2 MEFs on the coding region of tRA-inducible *Stra6* gene and the tRA-non inducible *Chordc1* gene. (E). Sequential native ChIP followed by DRIP on the promoter region of tRA-induced *Stra6* gene and the tRA-non induced *Chordc1* gene in untreated, tRA treated, UV exposed or UV exposed and tRA treated BirA and bXAB2 MEFs. (F). Sequential native ChIP followed by DRIP on the promoter region of tRA-induced *Sstr4* and *Fibin* intronless genes in untreated, tRA treated, UV exposed or UV exposed and tRA treated BirA and bXAB2 MEFs. (G) Sequential native ChIP followed by DRIP on the promoter region of tRA-induced *Stra6* and *Sstr4* (intronless) genes in tRA treated or UV exposed and tRA treated bXAB2, bXAB2;$Xpc^{-/-}$ or bXAB2;$Csb^{m/m}$ MEFs. DRIP /CHIP signals are shown, either as fold enrichment (F.E) of % input of antibody/bXAB2 over % input of control antibody (IgG)/BirA Error bars indicate S.E.M. among three biological replicates. All scatter and bar blots are presented as mean ± SEM. Error bars indicate S.E.M. among three biological replicates in all cases. *p* values were calculated by two tailed Student's t test, unless otherwise indicated. Scale bars, 15 μm, unless otherwise stated. Source data provided as a Source Data file.

Figure S6. UV irradiation leads to XAB2 release from the XPF-XPG R-loops dependent complex. (A). Representative images and quantification of RNA:DNA

hybrid immunofluorescence detection in bXAB2, bXAB2*;Xpc*$^{-/-}$ or bXAB2*;Csb*$^{m/m}$ MEFs tRA treated or UV exposed and tRA treated. Graph depicts mean S9.6 fluorescence intensity per nucleus (bXAB2 -/+UV: n=700/150 cells, bXAB2*;Xpc*$^{-/-}$ -/+UV: n=350/420 cells and bXAB2*;Csb*$^{m/m}$ -/+UV: n=770/780 cells/ 3 independent experiments in all cases) (). (B) bXAB2 RNA pull downs on U4 and U6 snRNAs in tRA or illudin-tRA treated BirA and bXAB2 MEFs transfected or not with RNase H. Error bars indicate S.E.M. among six biological replicates (C and F). bXAB2 pulldowns (PD: Pulldown) and western blot with anti-XPF, anti-XPG and anti-PRP19 or FLAG in nuclear extracts derived from untreated or UV exposed and tRA treated bXAB2 and BirA MEFs w/o RNaseH treatment. (D and E). bXPF pulldowns and western blot with anti-XPG, anti-XAB2 and FLAG in nuclear extracts derived from tRA treated or UV exposed and tRA treated bXPF and BirA MEFs w/o DNase treatment. (G and H). Western blot of RNA/DNA hybrid IP in nuclear extracts derived from untreated or UV exposed and tRA treated B6 MEFs w/o RNaseH treatment, using indicated antibodies. Isotype-matched IgG antibody were used as controls. (I). Cell cycle profiling and representative images of FACS analysis of wt. cells cultured in the presence (FBS+) or absence (FBS-) of fetal bovine serum. The images shown on Fig. S6 C-H are representative of experiments that were repeated three times. RIP signals are shown, as fold enrichment (F.E) of % input of bXAB2 over % input of BirA. , unless otherwise stated. All scatter and bar blots are presented as mean ± SEM. Error bars indicate S.E.M. among three biological replicates in all cases, unless otherwise stated. *p* values were calculated by two tailed Student's t test, unless otherwise indicated. Scale bars, 15 μm. Source data provided as a Source Data file.

Figure S7. XAB2 recruitment on R-loops does not require a functional NER. (A) Representative images and quantification of RNA:DNA hybrid immunofluorescence detection of wt. cells cultured in the presence (FBS+) or absence (FBS-) of fetal bovine serum transfected (RNH+) or not (RNH-) with RNaseH1 protein. Quantification depicts mean S9.6 fluorescence intensity per nucleus (-FBS -/+RNH: n=164/150 cells + FBS -/+ RNH: n= 180/173 cells/3 individual experiments)). (B). Western blot of RNA/DNA hybrid IP in nuclear extracts derived from tRA treated B6 MEFs w/o RNaseH treatment, using the indicated antibodies. Isotype-matched IgG antibody were used as controls. (C).Western blot of RNA:DNA hybrid IP in nuclear extracts derived from $Xpa^{-/-}$ MEFs, using the indicated antibodies. Isotype-matched IgG antibody were used as controls. (D). Representative images and quantification of RNA:DNA hybrid immunofluorescence detection of $Xpa^{-/-}$ cells. Graph depicts mean S9.6 fluorescence intensity per nucleus (wt: n=600 cells/3 independent experiments,: $Xpa^{-/-}$ n=550 cells/3 independent experiments)). (E). S9.6 DRIP analysis on the promoter regions of highly transcribed genes i.e. *Igf1* and *Ghr* w/o RNaseH1 (RNH) in wt. and $Csb^{m/m}$ P15 livers. (F). bXAB2 ChIP signals from BirA, bXAB2 and bXAB2;$Csb^{m/m}$ P15 livers on the promoter regions of highly transcribed genes (*Igf1* and *Ghr*). The images shown on Fig. S7 A-B are representative of experiments that were repeated three times. DRIP/CHIP signals are shown, either as fold enrichment (F.E) of % input of antibody over % input of control antibody (IgG) or as of % input of bXAB2 over % input of BirA. . Error bars indicate S.E.M. among three biological replicates, unless otherwise stated. All scatter and bar blots are presented as mean ± SEM. Error bars indicate S.E.M. among three biological replicates in all cases. *p*

values were calculated by two tailed Student's t test, unless otherwise indicated. Scale bars, 5 µm, unless otherwise stated. Source data provided as a Source Data file.

Supplementary Table 1: Oligonucleotide sequences

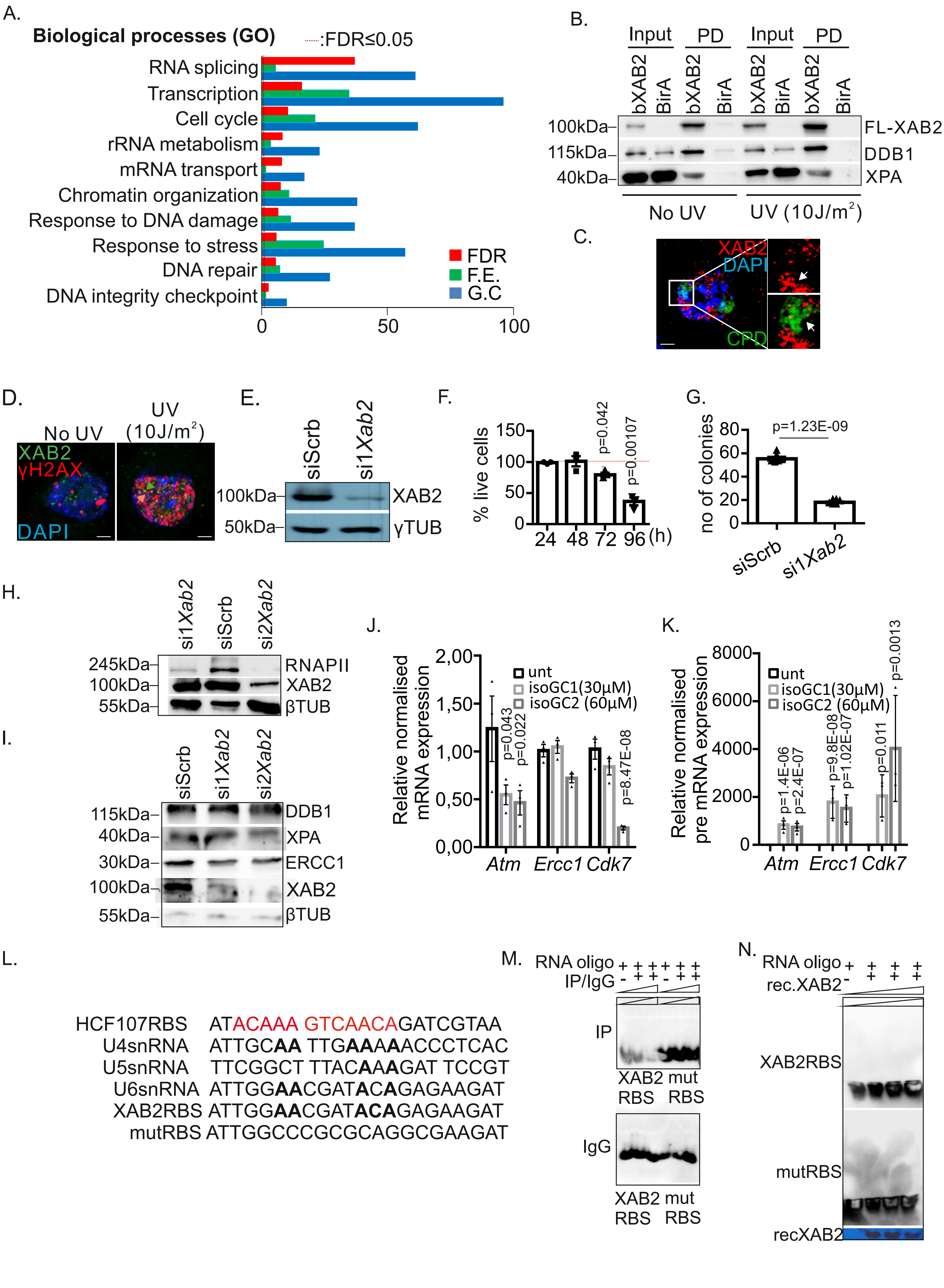

Goulielmaki et al., Fig. S1

A.

	Considered	Pdiff>0.8	Volcano (all non redudant)	Violin plots (IR, EX, Pdiff >0.8)	Differentially spliced Pdiff>0.8 and |ΔPSI|>5
IR	63180	849	63180	849	262
EX	22160	741	22160	741	358
Alt3'	23892	434	10957		89
Alt5'	17103	240	7752		65
Total	126335	2264	104049	1590	774

B.

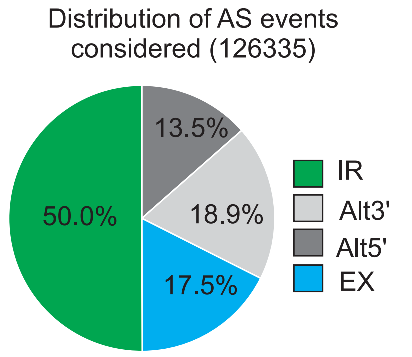

C.

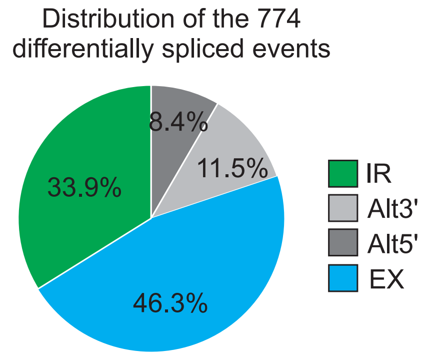

D.

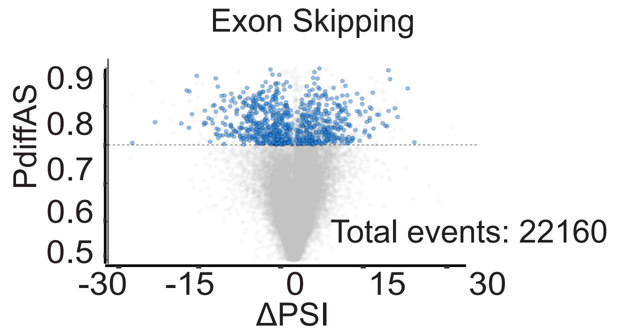

E.

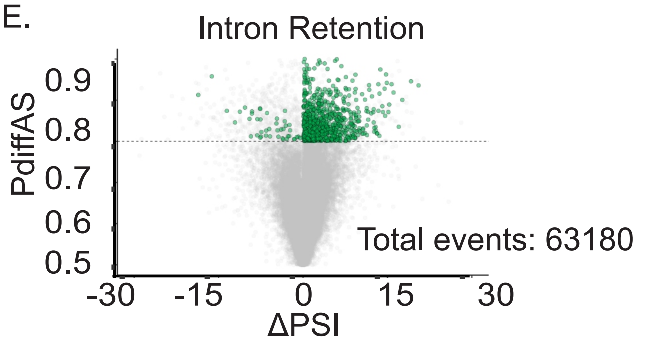

F.

	Considered	Pdiff>0.8	Volcano (all non redudant)	Violin plots (IR, EX, Pdiff >0.8)	Differentially spliced Pdiff>0.8 and |ΔPSI|>5
IR	28917	1558	28917	1558	748
EX	7468	347	7468	347	207
Alt3'	12156	412	5545		123
Alt5'	8136	182	3676		56
Total	56677	2499	45606	1905	1134

G.

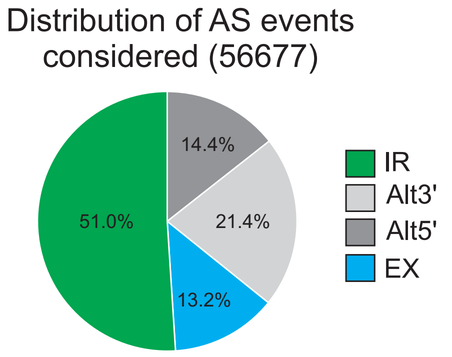

H.

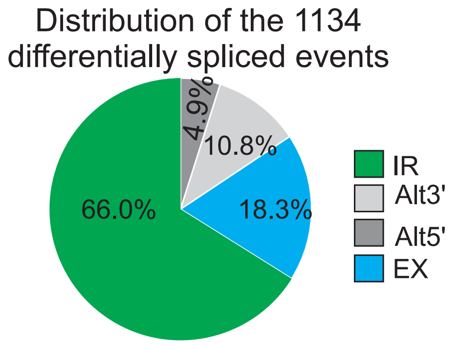

I.

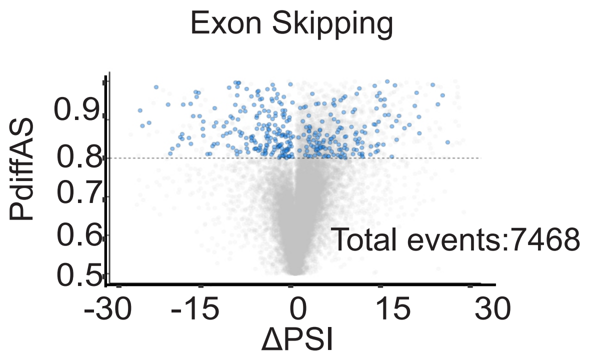

J.

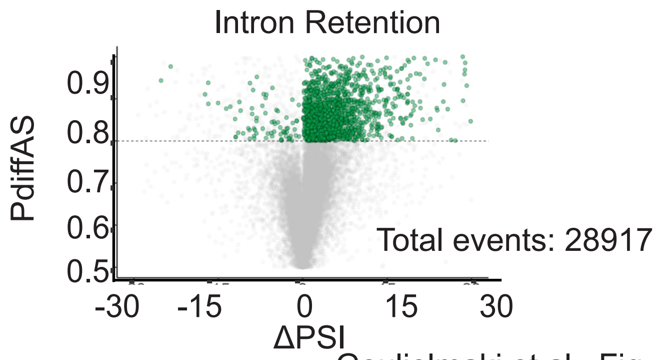

Goulielmaki et al., Fig. S2

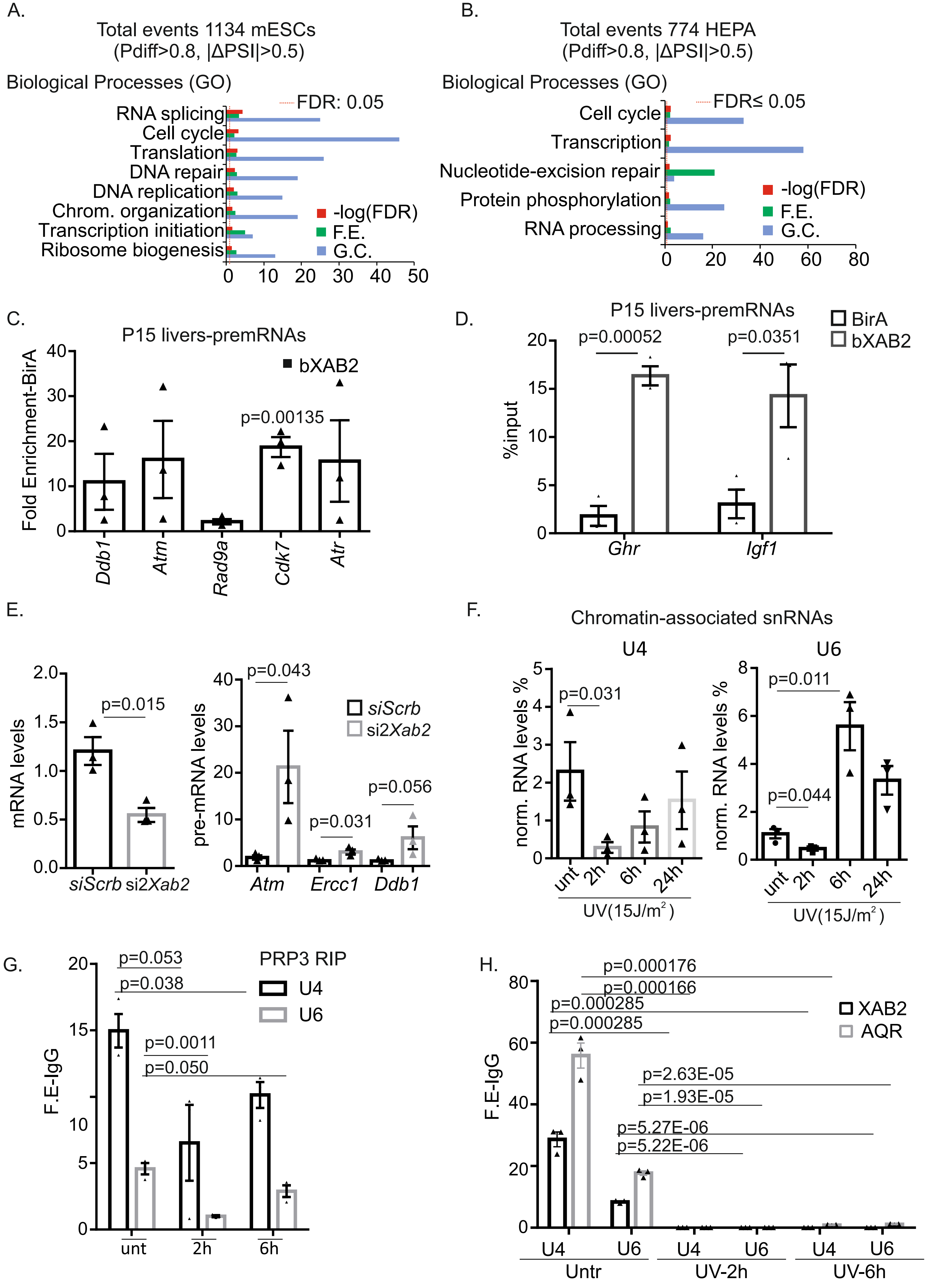

Goulielmaki et al., Fig. S3

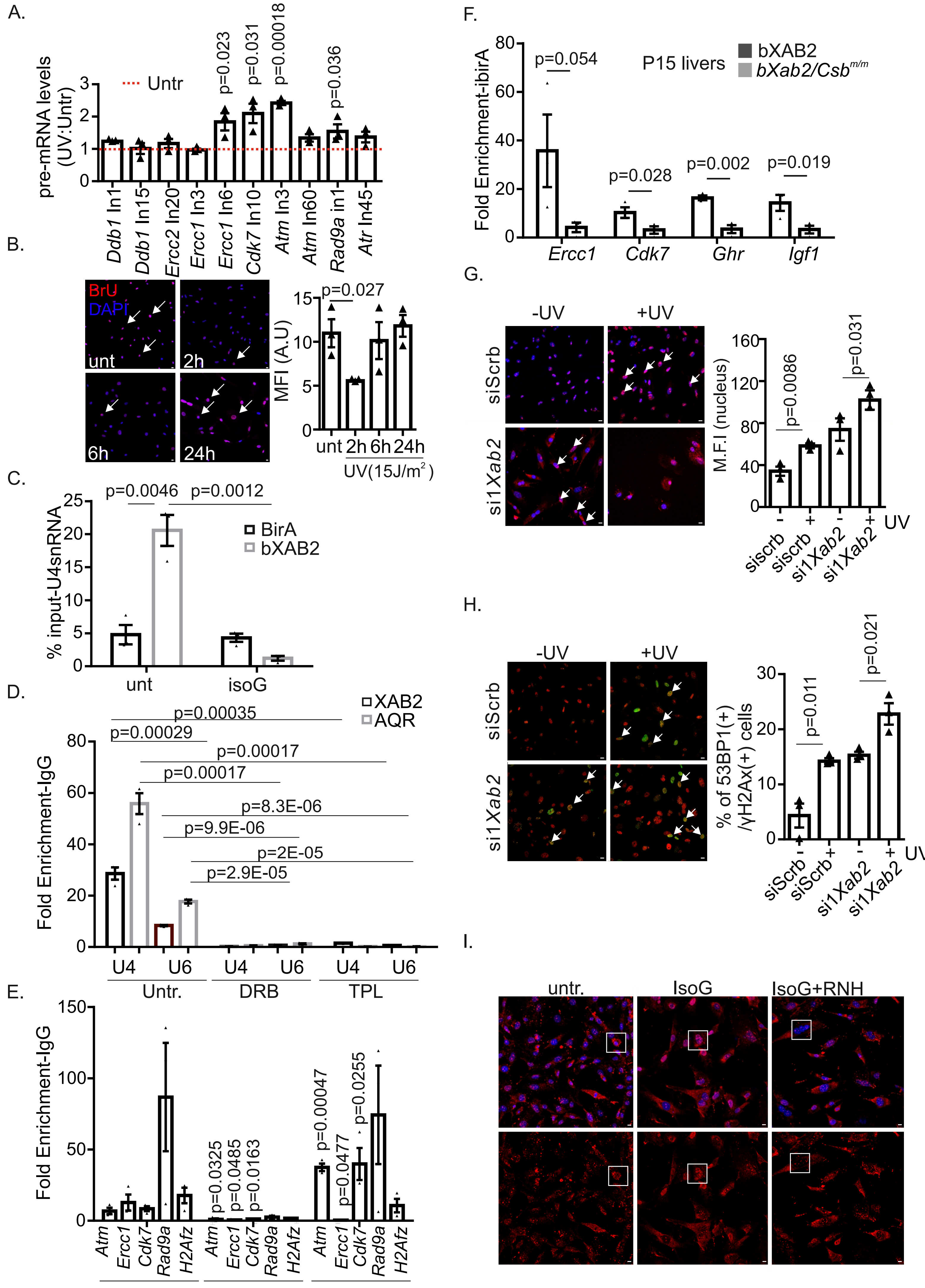

Goulielmaki et al., Fig. S4

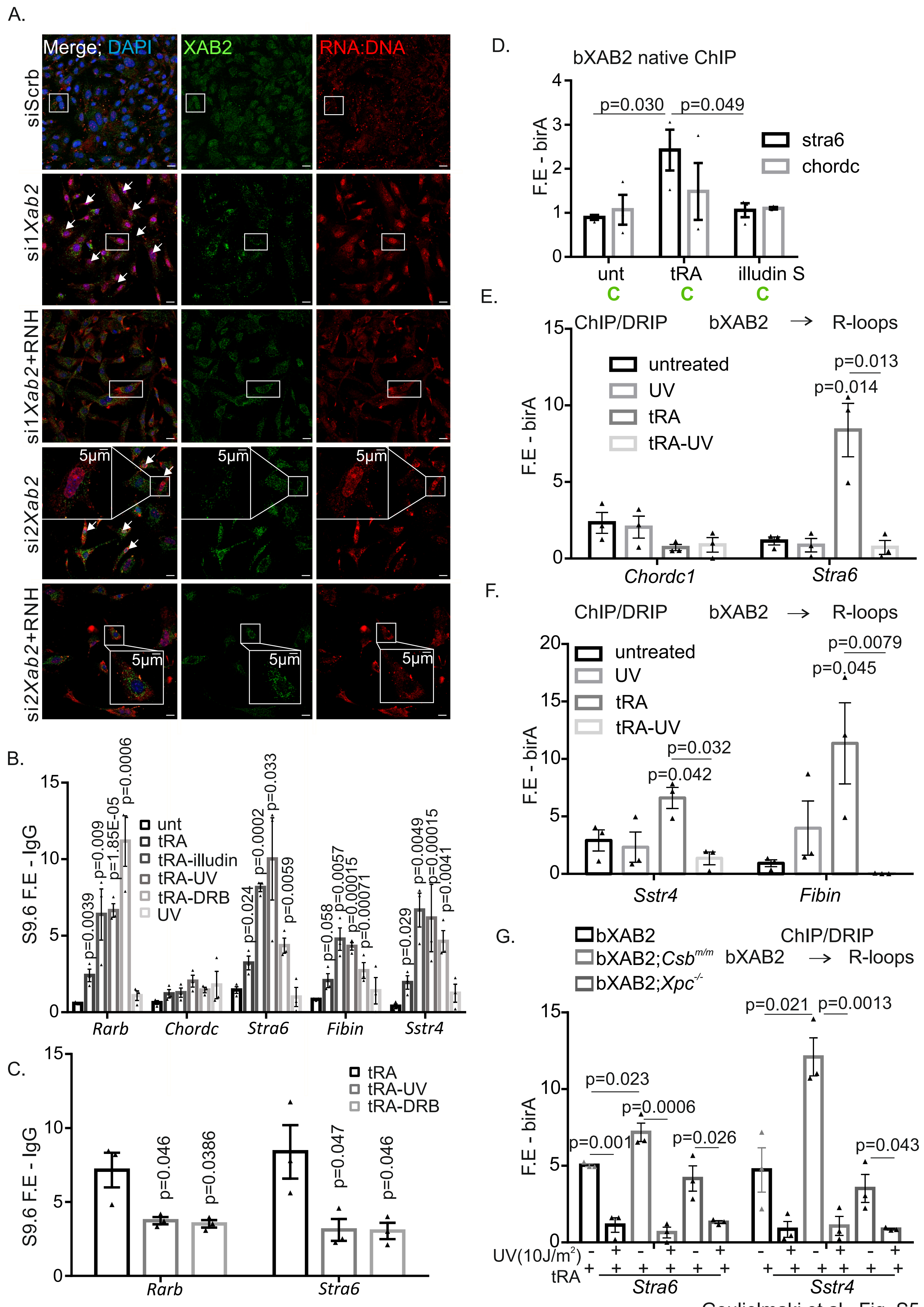

Goulielmaki et al., Fig. S5

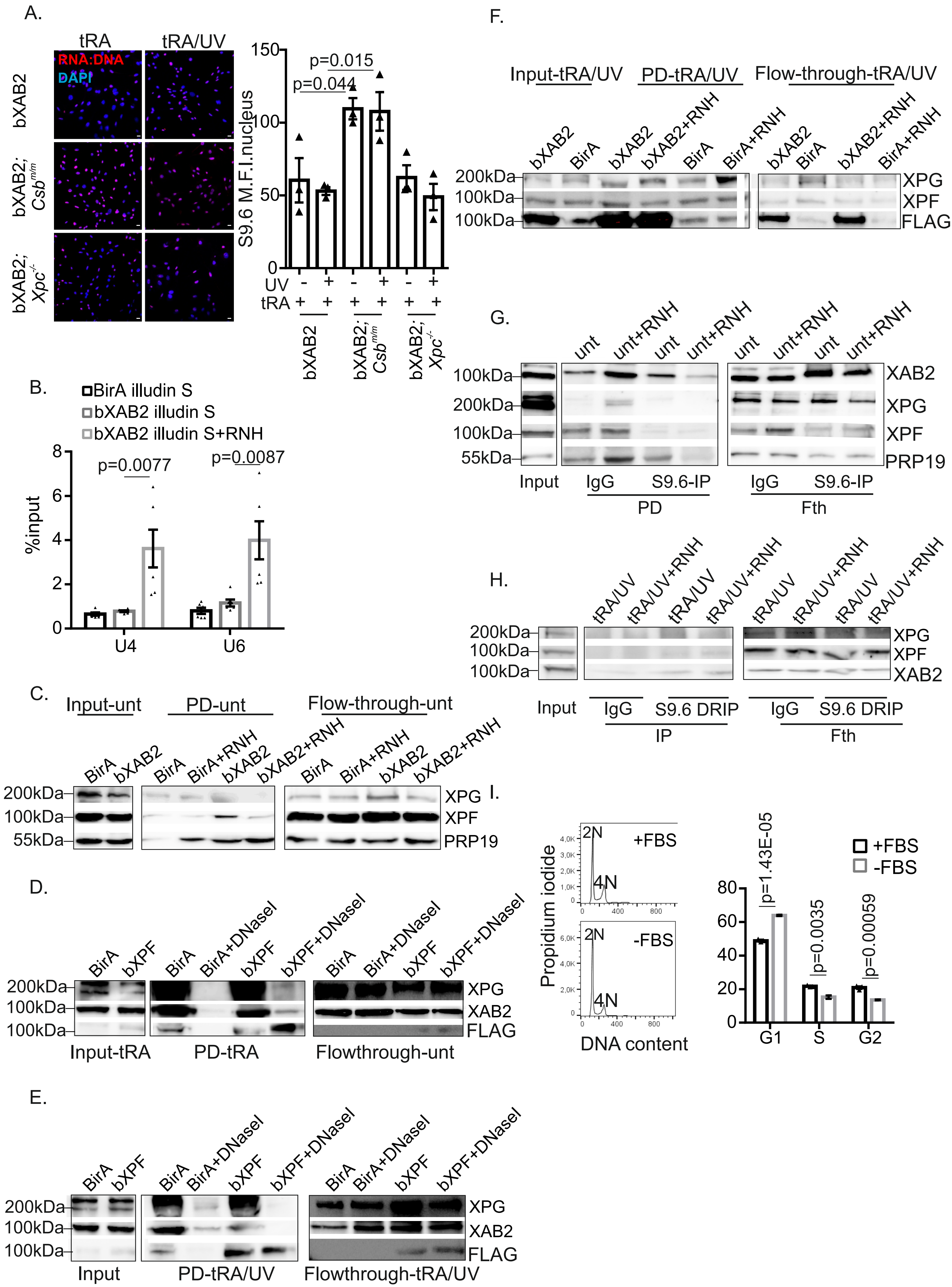

Goulielmaki et al., Fig. S6

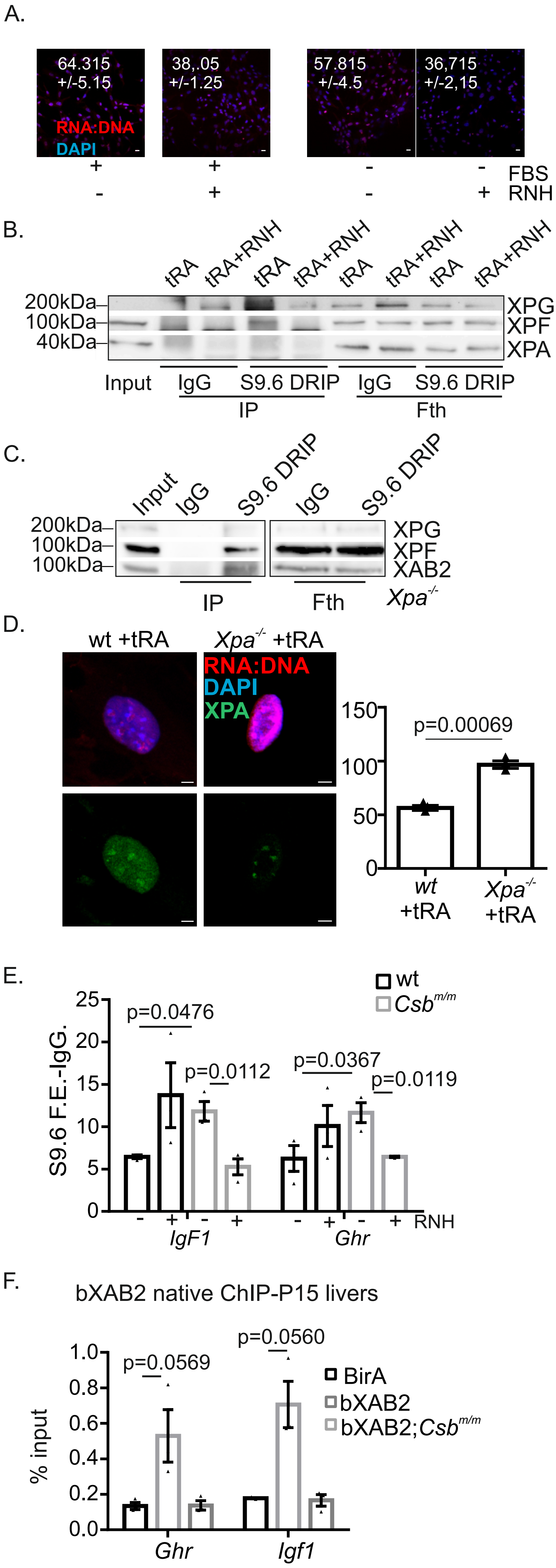

Goulielmaki et al., Fig. S7
