# Supplementary material for: The splicing factor XAB2 interacts with ERCC1-XPF and XPG for R-loop processing
Source: Nat Commun. 2021 May 26;12:3153. doi: 10.1038/s41467-021-23505-1 (PMC8155215; doi:10.1038/s41467-021-23505-1)
Supplement: Supplementary file 9 — Description of Additional Supplementary Files [file 41467_2021_23505_MOESM9_ESM.docx]

Description of additional supplementary information file

Title: Supplementary data 1.

Description: A list of 1167 bXAB2­bound proteins each files as Supplementary Movie/Audio/Data 1, etc. identified in the P15 bXAB2 biological replicates compared to BirA control livers. UP: Unique peptides.

Title: Supplementary data 2.

Description: A list of the 636 bXAB2­bound proteins identified in P15 livers shared across the three bXAB2 biological replicates compared to BirA control livers. UP: Unique peptides.

Title: Supplementary data 3.

Description: A list of 255 differentially expressed genes in HEPA cells transfected with dsRNA targeting the Xab2 transcript vs. scramble control cells. FC: Fold change.

Title: Supplementary data 4.

Description: A list of 333 differentially expressed genes in mESCs transfected with dsRNA targeting the Xab2 transcript vs. scramble control cells. FC: Fold change.

Tile: Supplementary data 5.

Description: Xab2 -induced differential splicing events in HEPA cells. COORD: Coordinates.

Title: Supplementary data 6.

Description: Xab2 -induced differential splicing events in mESCs. COORD: Coordinates.
