# Supplementary material for: The splicing factor XAB2 interacts with ERCC1-XPF and XPG for R-loop processing
Source: Nat Commun. 2021 May 26;12:3153. doi: 10.1038/s41467-021-23505-1 (PMC8155215; doi:10.1038/s41467-021-23505-1)
Supplement: Supplementary file 10 — Reporting Summary [file 41467_2021_23505_MOESM10_ESM.pdf]

nature research

Corresponding author(s): George A. Garinis

Last updated by author(s): Apr 13, 2021

Reporting Summary

Nature Research wishes to improve the reproducibility of the work that we publish. This form provides structure for consistency and transparency in reporting. For further information on Nature Research policies, see our Editorial Policies and the Editorial Policy Checklist.

Statistics

For all statistical analyses, confirm that the following items are present in the figure legend, table legend, main text, or Methods section.

n/a	Confirmed	
☐	☒	The exact sample size (n) for each experimental group/condition, given as a discrete number and unit of measurement
☐	☒	A statement on whether measurements were taken from distinct samples or whether the same sample was measured repeatedly
☐	☒	The statistical test(s) used AND whether they are one- or two-sided<br>*Only common tests should be described solely by name; describe more complex techniques in the Methods section.*
☒	☐	A description of all covariates tested
☒	☐	A description of any assumptions or corrections, such as tests of normality and adjustment for multiple comparisons
☒	☐	A full description of the statistical parameters including central tendency (e.g. means) or other basic estimates (e.g. regression coefficient) AND variation (e.g. standard deviation) or associated estimates of uncertainty (e.g. confidence intervals)
☐	☒	For null hypothesis testing, the test statistic (e.g. F, t, r) with confidence intervals, effect sizes, degrees of freedom and P value noted<br>*Give P values as exact values whenever suitable.*
☒	☐	For Bayesian analysis, information on the choice of priors and Markov chain Monte Carlo settings
☐	☒	For hierarchical and complex designs, identification of the appropriate level for tests and full reporting of outcomes
☐	☒	Estimates of effect sizes (e.g. Cohen's d, Pearson's r), indicating how they were calculated

Our web collection on statistics for biologists contains articles on many of the points above.

Software and code

Policy information about availability of computer code

Data collection: *Provide a description of all commercial, open source and custom code used to collect the data in this study, specifying the version used OR state that no software was used.*

Data analysis: *Provide a description of all commercial, open source and custom code used to analyse the data in this study, specifying the version used OR state that no software was used.*

For manuscripts utilizing custom algorithms or software that are central to the research but not yet described in published literature, software must be made available to editors and reviewers. We strongly encourage code deposition in a community repository (e.g. GitHub). See the Nature Research guidelines for submitting code & software for further information.

Data

Policy information about availability of data

All manuscripts must include a data availability statement. This statement should provide the following information, where applicable:

- Accession codes, unique identifiers, or web links for publicly available datasets
- A list of figures that have associated raw data
- A description of any restrictions on data availability

All data generated during this study are included in the published article (and its supplementary information files)

Field-specific reporting

Please select the one below that is the best fit for your research. If you are not sure, read the appropriate sections before making your selection.

☒ Life sciences ☐ Behavioural & social sciences ☐ Ecological, evolutionary & environmental sciences

For a reference copy of the document with all sections, see nature.com/documents/nr-reporting-summary-flat.pdf

Life sciences study design

All studies must disclose on these points even when the disclosure is negative.

Sample size	Sample size calculation was based on 1. effect size (the difference between the mean of two groups), 2. the standard deviation or standard error of the mean variability within the sample and 3. the decision of direction of effect (two tailed for all experiments performed).
Data exclusions	No data were excluded from the analysis.
Replication	All experiments were reproduced > 3 times (biological replicates) and in multiple technical replicates (at least 3 technical replicates).
Randomization	Samples were allocated to experimental groups according to genotypes or treatments.
Blinding	Investigators were not blinded to group allocations for all experiments performed in this work. This is due to the fact that the animal models used in this work have obvious phenotypes.

Reporting for specific materials, systems and methods

We require information from authors about some types of materials, experimental systems and methods used in many studies. Here, indicate whether each material, system or method listed is relevant to your study. If you are not sure if a list item applies to your research, read the appropriate section before selecting a response.

Materials & experimental systems

n/a	Involved in the study	
☐	☒	Antibodies
☐	☒	Eukaryotic cell lines
☒	☐	Palaeontology and archaeology
☐	☒	Animals and other organisms
☒	☐	Human research participants
☒	☐	Clinical data
☒	☐	Dual use research of concern

Methods

n/a	Involved in the study	
☒	☐	ChIP-seq
☒	☐	Flow cytometry
☒	☐	MRI-based neuroimaging

Antibodies

Antibodies used	Antibodies against XAB2 (wb: 1:2000, IF: 1:1000), PRP19 (wb: 1:1000, IF: 1:500), β-TUB (wb: 1:2000) were from Abcam. BCAS2 (wb: 1:5000, IF: 1:1000), DDB1 (IF: 1:500) were from Novus. HA (Y-11, western blotting (wb): 1:500), ERCC1 (D-10, wb: 1:500), RAD9A (wb: 1:300), POLII (wb: 1:500, IF: 1:50), XPA (wb: 1:500), XPF (F-11, wb: 1:500) and XPG (sc12558, wb: 1:200) were from Santa Cruz Biotechnology. γH2Ax (05-636, IF: 1:12.000) and S9.6 (MABE1095, IF: 1:300) was from Millipore. DDB1 (wb: 1:5000), XPC (wb: 1:1000) and CSB (wb: 1:1000) were from Bethyl Laboratories. Streptavidin-HRP (wb: 1:12,000) was from Upstate Biotechnology. FLAGM2 (F3165, wb 1:2.000) was from Sigma-Aldrich. CPD (IF: 1:50) was Cosmo Bio Ltd (TDM2). BrdU (1:300, IF) was from BD Pharmingen.
Validation	The validation of all primary antibodies was based on available references in the manuscript and information provided on the manufacturer website and associated literature.

Eukaryotic cell lines

Policy information about cell lines

Cell line source(s)	All primary cells were derived from bXAB2, NER mutant or wild-type animal models (as indicated in the manuscript).
Authentication	The genotype of primary cells was authenticated by PCR and/or sequencing and western blotting (when appropriate).
Mycoplasma contamination	All primary cells were tested negative for mycoplasma.

Commonly misidentified lines (See ICLAC register)	We have not used any misidentified lines in this manuscript.

Animals and other organisms

Policy information about studies involving animals; ARRIVE guidelines recommended for reporting animal research

Laboratory animals	Mus musculus; strain: Bl/6; sex: males or females (as indicated).
Wild animals	There was no use of wild animals.
Field-collected samples	There is no work with field-collected samples.
Ethics oversight	Foundation of Research and Technology Hellas

Note that full information on the approval of the study protocol must also be provided in the manuscript.
